# Dual role for Fcγ receptors in host defense and disease in *Borrelia burgdorferi*-infected mice

**DOI:** 10.3389/fcimb.2014.00075

**Published:** 2014-06-11

**Authors:** Alexia A. Belperron, Nengyin Liu, Carmen J. Booth, Linda K. Bockenstedt

**Affiliations:** ^1^Section of Rheumatology, Department of Internal Medicine, Yale University School of MedicineNew Haven, CT, USA; ^2^Section of Comparative Medicine, Yale University School of MedicineNew Haven, CT, USA

**Keywords:** Fc receptor, *Borrelia burgdorferi*, arthritis, mice, Lyme disease, toll-like receptors, MyD88

## Abstract

Arthritis in mice infected with the Lyme disease spirochete, *Borrelia burgdorferi*, results from the influx of innate immune cells responding to the pathogen in the joint and is influenced in part by mouse genetics. Production of inflammatory cytokines by innate immune cells *in vitro* is largely mediated by Toll-like receptor (TLR) interaction with Borrelia lipoproteins, yet surprisingly mice deficient in TLR2 or the TLR signaling molecule MyD88 still develop arthritis comparable to that seen in wild type mice after *B. burgdorferi* infection. These findings suggest that other, MyD88-independent inflammatory pathways can contribute to arthritis expression. Clearance of *B. burgdorferi* is dependent on the production of specific antibody and phagocytosis of the organism. As Fc receptors (FcγR) are important for IgG-mediated clearance of immune complexes and opsonized particles by phagocytes, we examined the role that FcγR play in host defense and disease in *B. burgdorferi*-infected mice. *B. burgdorferi*-infected mice deficient in the Fc receptor common gamma chain (FcεR_γ^−/−^_ mice) harbored ~10 fold more spirochetes than similarly infected wild type mice, and this was associated with a transient increase in arthritis severity. While the elevated pathogen burdens seen in *B. burgdorferi*-infected MyD88^−/−^ mice were not affected by concomitant deficiency in FcγR, arthritis was reduced in FcεR_γ^−/−^_MyD88^−/−^ mice in comparison to wild type or single knockout mice. Gene expression analysis from infected joints demonstrated that absence of both MyD88 and FcγR lowers mRNA levels of proteins involved in inflammation, including Cxcl1 (KC), Xcr1 (Gpr5), IL-1beta, and C reactive protein. Taken together, our results demonstrate a role for FcγR-mediated immunity in limiting pathogen burden and arthritis in mice during the acute phase of *B. burgdorferi* infection, and further suggest that this pathway contributes to the arthritis that develops in *B. burgdorferi*-infected MyD88^−/−^ mice.

## Introduction

Lyme disease, due to infection with *Borrelia burgdorferi sensu lato* spirochetes, is the most common arthropod-borne disease in the northern hemisphere (Kurtenbach et al., 2006). The disease usually manifests as the skin lesion erythema migrans at the tick bite site and can result in myocarditis, arthritis, and neurological abnormalities once infection has disseminated (Bockenstedt, 2013). In the United States, arthritis is the second most common clinical sign of confirmed cases of Lyme disease reported to the Centers for Disease Control and Prevention (CDC, 2013). A mouse model of Lyme disease has been developed that manifests primarily as myocarditis and arthritis, although spirochetes can be found in other, non-diseased tissues such as the skin (Barthold et al., 1992). Inflammation peaks in the heart and joints 2–4 weeks after infection introduced by needle inoculation of cultured spirochetes and resolves without treatment in wild type mice even though spirochetes remain in these tissues (Barthold et al., 1990). Histopathology of *B. burgdorferi*-infected mouse tissues reveals a dominance of innate immune cells at sites of inflammation, especially macrophages in the heart and neutrophils in the joints (Barthold et al., 1990, 1992; Ruderman et al., 1995). This pathology develops in the absence of B and T cells, but adaptive immunity is required for the spontaneous, immune-mediated regression of disease (Barthold et al., 1992; McKisic and Barthold, 2000; Bockenstedt et al., 2001).

*B. burgdorferi* express an abundant array of lipoproteins that are potent stimulators of inflammatory responses (Fraser et al., 1997). Lipoproteins activate macrophages and other innate immune cells through Toll-like receptor (TLR) pattern recognition molecules, especially TLRs 2, 5, 7, 8, and 9 (Aliprantis et al., 1999; Hirschfeld et al., 1999; Wooten et al., 2002; Shin et al., 2008; Petzke et al., 2009; Cervantes et al., 2011). These TLRs utilize the common intracellular adaptor molecule myeloid differentiation primary response gene 88 (MyD88) to initiate intracellular signaling events that culminate in NFκb activation and inflammatory cytokine production (Creagh and O'Neill, 2006). *In vitro* stimulation of macrophages by borrelia lipoproteins via TLR2 leads to the production of chemokines, cytokines, and the upregulation of costimulatory molecules (Hirschfeld et al., 1999; Shin et al., 2008; Salazar et al., 2009). While the TLR/MyD88 signaling pathway is the main pathway contributing to *B. burgdorferi* lipoprotein mediated inflammation *in vitro*, arthritis and carditis still develop *in vivo* in the absence of TLR2 or MyD88 expression (Wooten et al., 2002; Liu et al., 2004; Behera et al., 2006b). This observation suggests that other MyD88-independent pathways contribute to the development of disease.

Receptors for the Fc region of IgG (FcγR) and immune complexes can also play a role in the development of inflammation (Nimmerjahn and Ravetch, 2006, 2008). FcγR couple innate and adaptive immune responses through their ability to activate effector cells (Nimmerjahn and Ravetch, 2008). Proinflammatory and anti-inflammatory mechanisms are linked to different FcγR, which share the common FcR γ-chain (Nimmerjahn and Ravetch, 2006). The high affinity FcγRI, intermediate affinity FcγRIV, and low affinity FcγRIII and Fcε RI, which mediate the binding and internalization of mouse IgG1, IgG2a, IgG2b, IgG3, and IgE subclasses, are activating receptors that contain an immunoreceptor tyrosine-based activation motif (ITAM) (Nimmerjahn and Ravetch, 2007). FcγRII is an inhibitory receptor containing an immunoreceptor tyrosine-based suppression motif. FcγR are predominantly expressed on macrophages, neutrophils, dendritic cells, and other innate immune cells, and have limited expression on lymphocytes, including B cells, and NK cells and endothelial cells (Takai, 2005). The outcome from engagement of FcγR depends on the IgG subclass and the balance between activation and inhibitory FcγR stimulation.

Mice deficient in the common gamma chain (FcεR_γ^−/−^_ mice) lack the ability to mediate IgG dependent phagocytosis and antibody-dependent cell-mediated cytotoxicity through FcγRI, FcγRIII, and FcγRIV (Takai et al., 1994). In some infection models, the ability to control infection is impaired in FcεR_γ^−/−^_ mice, as has been reported for intracellular pathogens *Plasmodium* and *Pneumocystis* spp. (Yoneto et al., 2001; Wells et al., 2006). Yet, with other infections and with some autoimmune models, the absence of FcγR signaling ameliorates disease (Kima et al., 2000; Tarzi et al., 2002; Alexander and Scott, 2004; Kaneko et al., 2006a,b; Giorgini et al., 2008). Antigen-antibody complexes (immune complexes) can trigger inflammation by binding to and activating FcγR, and these receptors have been shown to play an integral role in immune complex-mediated tissue injury (Jancar and Crespo, 2005). When antigens encounter their cognate antibody in the presence of complement, binding of the complex to the complement C3 protein follows immune complex formation (Wessels et al., 1995; Baudino et al., 2008; Giorgini et al., 2008). This complex facilitates simultaneous interaction with both FcγR and complement receptors. Activation of complement can lead to clearance of pathogens as well as the immune complexes, thereby preventing them from causing further tissue damage. More recently it has been found that signaling through both FcγR and TLRs are modulated by ITAM-coupled receptors. ITAM receptors are activated by immune complexes that crosslink FcγR, and this high avidity activation synergizes with TLR signals to activate pro-inflammatory gene expression (Ivashkiv, 2008).

Immune complexes containing *B. burgdorferi* antigens have been found early after infection in human plasma samples (Benach et al., 1984; Schutzer et al., 1999; Lencáková et al., 2007). Here, we postulated that inflammation in *B. burgdorferi-infected* mice could be mediated in part by the formation of immune complexes, and that this FcγR-mediated inflammation may contribute to the pathology seen in mice deficient in TLR/MyD88-mediated responses. Our results using mice deficient in FcγR only or in both FcγR and MyD88 reveal that FcγR has a dual role in immune defense and arthritis development associated with *B. burgdorferi* infection.

## Methods

### Mice

C57Bl/6 (B6);129P2-Fcer1^*gtm1Rav*^/J (FcεR_γ^−/−^_) mice, 6–8 weeks of age, were obtained from the Jackson Laboratory (Bar Harbor, ME, stock number 002847). The B6.129 MyD88^−/−^ mice were the kind gift of Ruslan Medzhitov, Yale University School of Medicine (Schnare et al., 2001). FcεR_γ^−/−^_ mice were bred with MyD88^−/−^ mice and select progeny were intercrossed to produce FcεR_γ^−/−^_ MyD88^−/−^ double knockout mice; the F2 generation was used in these studies. Wild type and heterozygous littermates were used as controls. Mice were housed in filter frame cages and administered food and water *ad libitum* according to Yale University animal care and use guidelines. Mice were euthanized by carbon dioxide asphyxiation. The Yale University Institutional Animal Care and Use Committee approved all procedures.

### Infection of mice

Frozen aliquots of low-passage *B. burgdorferi* N40 were grown to mid-logarithmic phase in BSK-H medium (Sigma-Aldrich, St. Louis, MO) and enumerated by dark-field microscopy using a Petroff-Hausser chamber. Each mouse was inoculated intradermally in the central lower back or in each hind limb between the knee and tibiotarsal joints with 10^4^ spirochetes in 100 μl of BSK-H medium. These inoculation sites were chosen as we have found that arthritis may be more apparent in a disease-resistant mouse background such as C67BL/6 or 129 mice if spirochetes are inoculated closer to the joints examined for histopathology. Both inoculation sites still result in systemic disseminated infections. At the time of mouse sacrifice, infection was confirmed by culturing the blood or a portion of the urinary bladder in BSK-H medium for 14 days, after which the presence of spirochetes was determined by dark-field microscopy.

### Macrophage stimulation

Macrophages were isolated from WT and MyD88^−/−^ mice by peritoneal lavage according to previously published methods (Liu et al., 2004). Cells were resuspended in α-minimal essential medium (MEM) supplemented with 10% heat-inactivated fetal bovine serum and aliquoted at 2 × 10^6^ cells/ml into a 24-well plate. Cells were stimulated for 24 h with recombinant lipidated OspC protein (kindly provided by John Dunn, Brookhaven National Laboratories) or Osp C protein-immune complexes formed using rabbit polyclonal anti-OspC antisera. We confirmed that antibodies present in the rabbit antisera could bind to mouse FcγR using the J774 mouse macrophage cell line (data not shown). In order to create the immune complexes, concentrations of lipidated OspC protein ranging from 0.1 ng to 1 μ g were incubated with 20 μ l of rabbit polyclonal anti-OspC antisera (with an anti-OspC IgG titer of 1:192,000) and 80 μ l of α-MEM media for 30 min prior to incubation with the cells. Negative control samples contained just the rabbit polyclonal anti-OspC antisera in media to insure that antisera alone did not stimulate production of TNFα. Cells were cultured for 24 h in a 37°C incubator with 5% CO_2_, after which supernatants were harvested and analyzed for TNFα production by ELISA as described (Liu et al., 2004).

### Measurement of *B. burgdorferi*-specific antibody titers

*B. burgdorferi*-specific enzyme-linked immunosorbent assays were performed using sera from 21-day infected animals as previously described (Liu et al., 2004). Briefly, 96-well microtiter plates were coated with *B. burgdorferi* lysate (3 μg in 50 μl 100% ethanol per well). After blocking, serial twofold dilutions of sera in PBS containing 0.5% bovine serum albumin and 0.5% Tween 20 were added to the wells and incubated for 1 h at room temperature. Secondary biotinylated anti-mouse IgM and IgG (Vector Labs, Burlingame, CA), IgG1, IgG2a, and IgG2b (Invitrogen, Carlsbad, CA), and IgG3 (BD Biosciences, Franklin Lakes, NJ) were used at a 1:1000 dilution, and bound Abs were detected with the ABC Elite peroxidase detection and ABTS [2,2′-azino-bis(ethylbenzthiazolinesulfonic acid)] substrate kits (Vector Labs). The mixed B6.129 mouse background permitted reactivity with IgG2a even though the B6 mouse strain lacks this subclass and expresses the IgG2c subclass instead (with 70% homology to IgG2a).

### Quantitative PCR of *B. burgdorferi* DNA

DNA was isolated from urinary bladder using the DNeasy kit (QIAGEN Inc., Valencia, CA) according to the manufacturer's instructions. This tissue was chosen as representative of pathogen burden as the entire organ can be processed for DNA extraction and, as a non-diseased site, the immune response would not be expected to impact the spirochete quantification. The copy number of *B. burgdorferi* in each sample was determined by quantitative PCR of the *B. burgdorferi recA* gene using an iCycler (Bio-Rad, Hercules, CA), the Brilliant SYBR green kit (Stratagene, Cedar Creek, TX), and the following primers: 5′ primer 5′-GTGGATCTATTGTATTAGATGAGGCTCTCG-3′ and 3′ primer 5′-GCCAAAGTTCTGCAACATTAACACCTAAAG-3′. The *recA* copy number was normalized to the mouse *actin* gene amplified using the following primers and probe: 5′ primer 5′-ATCAGGTAGTCGGTCAGG-3′, 3′ primer 5′-GGTATCTATCTCGACTC-3′, and probe 6-carboxyfluorescein-TCCAGCAGATCTGGATCAGCAAGCA-carboxytetra-methylrhodamine (Applied Biosystems, Foster City, CA). A 60°C annealing temperature and 45 or 50 cycles were used for the *recA* and *actin* gene reactions, respectively. Standard curves were generated for both PCRs using known quantities of DNA. Reactions were performed in duplicate, and the quantities of PCR products generated were determined from the standard curves.

### Histopathology

Bilateral hind limb joints (knee and tibiotarsal joints) were fixed in formalin, embedded in paraffin and sections stained with hematoxylin and eosin by routine histologic techniques as previously described (Liu et al., 2004). The knee and tibiotarsal joints were scored for the presence and severity of periarticular inflammation and arthritis on a scale of 0 (negative) to 3 (severe) in a blinded fashion as described previously (Liu et al., 2004). Values for arthritis severity were reported as the mean scores of all the joints of individual mice in each group ± the standard error of the mean. Carditis was considered present when inflammatory cell infiltrates were present in the heart base in tissue sections.

### Quantitative real-time array analysis for inflammatory cytokines and receptors

RNA was extracted from joints of uninfected and 21 day-infected WT, FcεR_γ^−/−^_, MyD88^−/−^and double knockout mice using TRIzol® and the PureLink™ micro-to-midi total RNA purification system (Invitrogen, Carlsbad, CA). The quality of RNA was verified in all samples with A_260_:A_230_ ratios greater than 1.7, and A_260_:A_280_ ratios between 1.8 and 2.0. RNA was combined from 4 mice in each group and was converted to complimentary DNA (cDNA) and real-time PCR was performed on the samples using the RT^2^ First Strand Kit and the RT^2^ qPCR Master Mix according the manufacturer's instructions (SABiosciences, Frederick, MD). The master mix containing the cDNA was loaded onto the RT^2^ Profiler PCR Array for Inflammatory Cytokines and Receptors (SABiosciences, PAMM-011), and the amplification was performed according to the manufacturer's protocol using a Bio-Rad iCycler. The data were analyzed using the ΔΔCt method by the Keck facility (Yale University).

### PCR confirmation of select microarray data

0.4 μ l aliquots of cDNA (generated above) were used as templates for PCR reactions in order to confirm select results obtained with the microarray. A 60°C annealing temperature and 35 cycles were performed for each reaction using Taq polymerase (QIAGEN) and the following primers: TNFα, 5′ primer 5′atgagcacagaaagcatgatc 3′ and 3′primer 5′tacaggcttgtcaccgaatt 3′; IL-10, 5′ primer 5′atgcaggactttaagggttacttg3′ and 3′ primer 5′tagacaccttggtcttggagctta 3′; IFNγ, 5′ primer 5′gacagaagttctgggcttctcc 3′ and 3′ primer 5′gcagcgactccttttccgctt 3′.

## Results

### Presentation of *B. burgdorferi* antigens as immune complexes enhances macrophage production of TNFα

**Figure 1:**
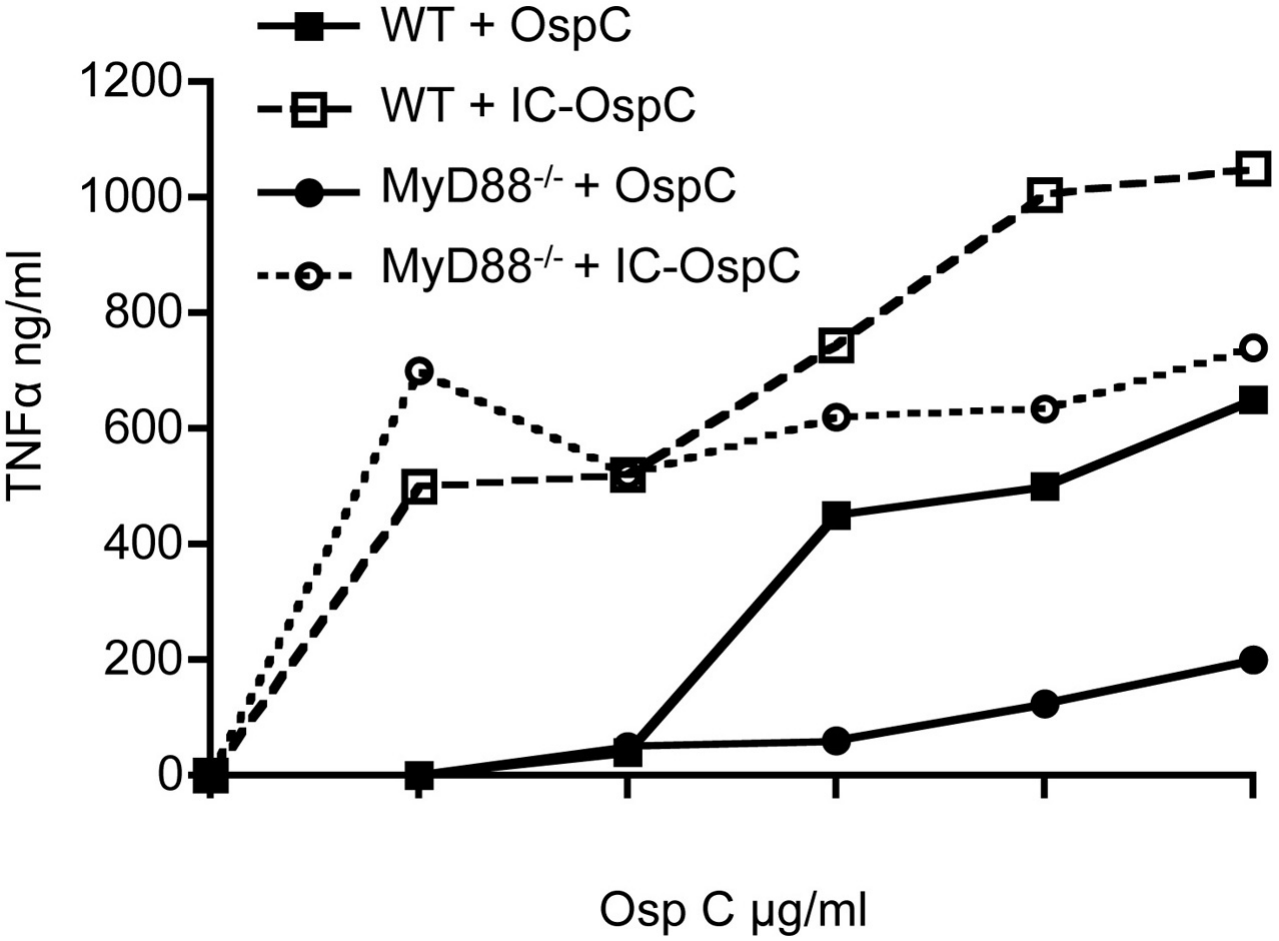
**TNFα secretion by macrophages stimulated with OspC and OspC-immune complexes**. Peritoneal macrophages from naïve WT and MyD88^−/−^ mice were stimulated *in vitro* with recombinant *B. burgdorferi* OspC protein or OspC-anti-OspC immune complexes for 24 h. The levels of secreted TNFα in the supernatants were measured by ELISA.

To examine the response of mouse macrophages to a *B. burgdorferi* antigen alone or in the form of an immune complex, we stimulated resting peritoneal macrophages from WT or MyD88^−/−^ mice with recombinant lipidated OspC alone or as an immune complex with anti-OspC antisera (Figure 1). As expected, recombinant lipidated OspC elicited TNFα in a dose-dependent fashion from WT macrophages, whereas there was little response of MyD88^−/−^ macrophages to this stimulation. In the presence of OspC antibody, however, the dose of OspC required to stimulate TNFα from WT macrophages was reduced 100-fold, from 0.01 to 0.0001 μg/ml (Figure 1). MyD88^−/−^ macrophages also produced TNFα when stimulated with OspC as an immune complex, at levels approaching those elicited from WT cells. OspC immune serum alone had no effect (data not shown). These findings indicate that in the absence of MyD88, macrophages can produce inflammatory cytokines when *B. burgdorferi* antigens are presented as immune complexes.

### FcγR deficiency transiently increases pathogen burdens early after *B. burgdorferi* infection

**Figure 2:**
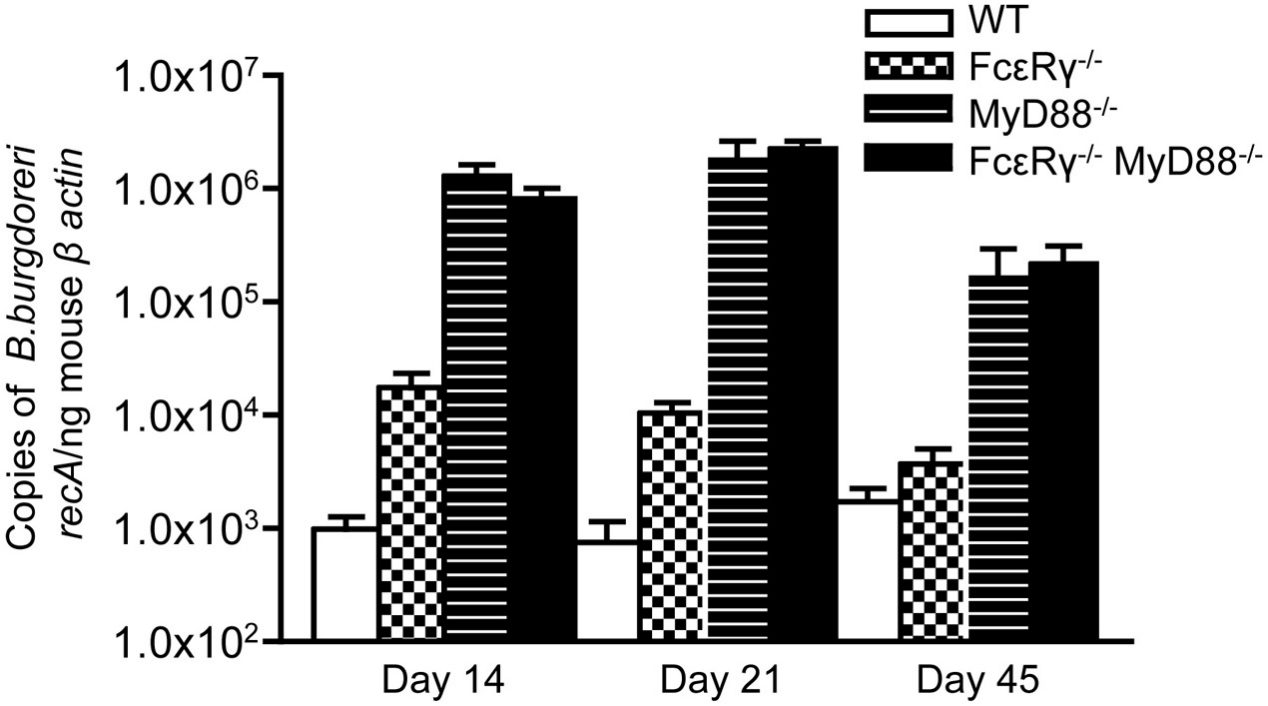
**Quantitative PCR of *B. burgdorferi* in urinary bladders of infected mice**. The copy number of the *B. burgdorferi recA* gene was normalized to the mouse β-actin gene by real-time PCR. Values are averages of 5–11 individual mice. Results are combined from 2 separate experiments. At day 14, the burdens in the FcεR_γ^−/−^_ mice are statistically different from those in the WT mice (*P* = 0.0043) and those in the MyD88^−/−^ and FcεR_γ^−/−^_ MyD88^−/−^ mice (*P* = 0.0029 and *P* = 0.0095, respectively). At day 21 the FcεR_γ^−/−^_ mice maintain statistically higher burdens than the WT mice (*P* = 0.007) but lower burdens than the MyD88^−/−^ (*P* = 0.001) and the FcεR_γ^−/−^_ MyD88^−/−^ (*P* ≤ 0.0001). At day 45 there is no difference between the WT and FcεR_γ^−/−^_ mice but the MyD88^−/−^ and FcεR_γ^−/−^_ MyD88^−/−^ burdens remain high. All *P*-values were calculated using the Mann-Whitney assay.

We next sought to determine the effects of FcγR deficiency on *B. burgdorferi* infection and disease *in vivo*. Although opsonization of *B. burgdorferi* is not required for its uptake by macrophages *in vitro* (Montgomery et al., 1994), FcγR could contribute to limiting pathogen burden by enhancing the uptake and lysosomal targeting of opsonized *B. burgdorferi*. We found that FcεR_γ^−/−^_ mice have 10-fold higher pathogen burdens as compared to WT mice at day 14 after infection (Figure 2, checkered bars vs. open bars). MyD88^−/−^ macrophages are known to have impaired uptake and degradation of *B. burgdorferi in vitro* (Liu et al., 2004; Behera et al., 2006a) and MyD88^−/−^ mice exhibit elevated pathogen burdens after *B. burgdorferi* infection (Bolz et al., 2004; Liu et al., 2004). As expected, the pathogen burdens in mice deficient in MyD88 were about 100-fold higher than those in WT mice (Figure 2, hatched bars), and combined deficiency in both FcγR and MyD88 did not lead to a further increase (hatched bars vs. solid black bars). Pathogen burdens remained elevated in FcεR_γ^−/−^_ mice through infection day 21, but by day 45, levels were comparable to those seen in WT mice. In contrast, MyD88^−/−^ and FcεR_γ^−/−^_ MyD88^−/−^ still had significantly elevated pathogen burdens at this late time point (Figure 2).

### Deficiency in FcγR reduces the severity of disease in MyD88^−/−^ mice

**Figure 3:**
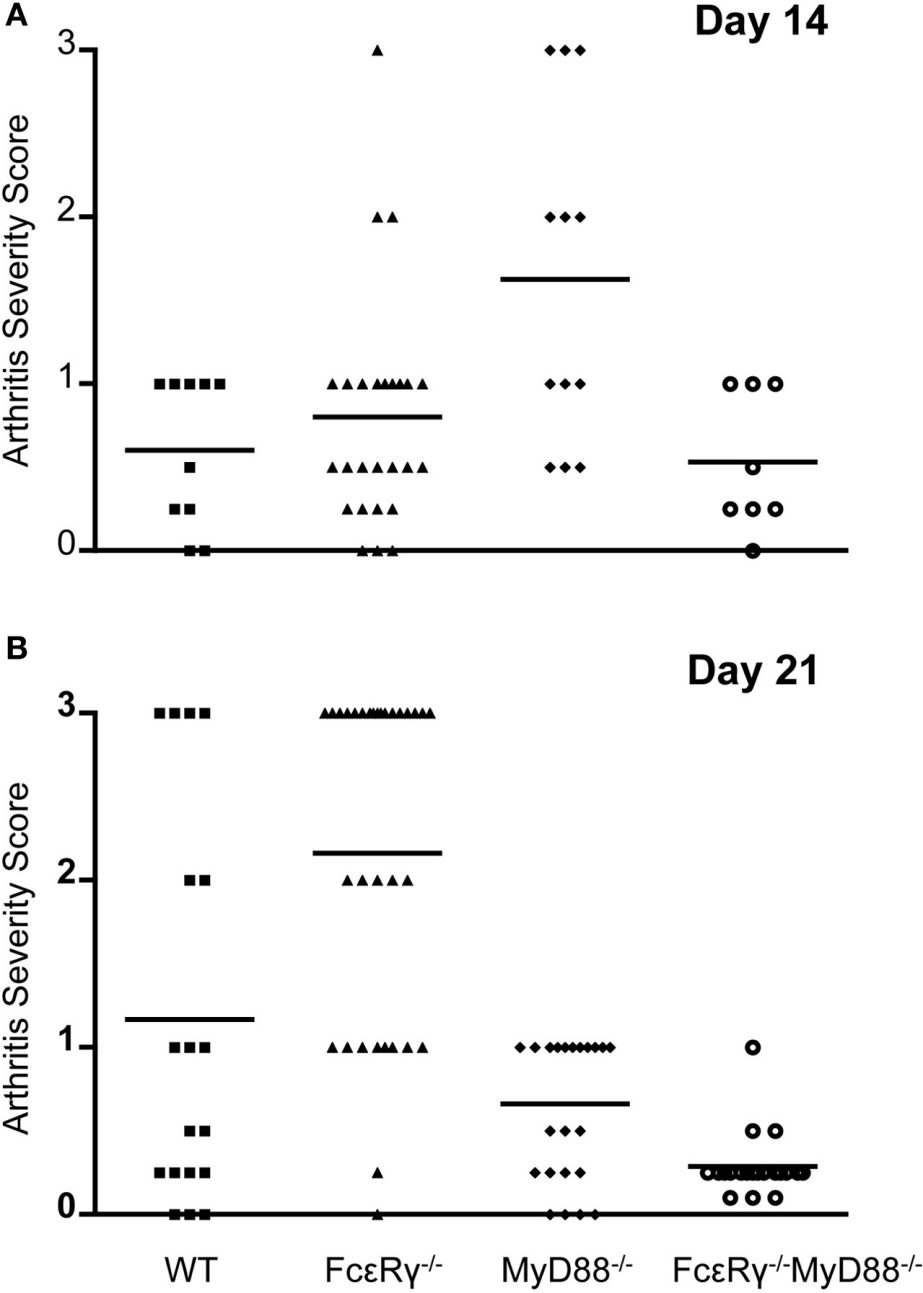
**Arthritis severity is diminished in the combined absence of both FcγR and MyD88**. Hind limb joints were analyzed, and each data point represents one joint. Arthritis was scored on a scale of 0 (negative) to 3 (severe). Four to 14 mice were used in each group and results were combined from 2 to 4 separate experiments. **(A)** shows arthritis severity at day 14, and **(B)** shows arthritis severity at day 21. Significant differences were observed in **(A)**: WT vs. MyD88^−/−^ *P* = 0.007, FcεR_γ^−/−^_ vs. MyD88^−/−^ *P* = 0.004, and MyD88^−/−^ vs. FcεR_γ^−/−^_ MyD88^−/−^ *P* = 0.009. **(B)** FcεR_γ^−/−^_ vs. WT *P* = 0.0026, FcεR_γ^−/−^_ vs. MyD88^−/−^ *P* ≤ 0.0001, FcεR_γ^−/−^_ vs. FcεR_γ^−/−^_ MyD88^−/−^*P* ≤ 0.0001, MyD88^−/−^ vs. FcεR_γ^−/−^_ MyD88^−/−^*P* = 0.0003, and WT vs. FcεR_γ^−/−^_ MyD88^−/−^ *P* = 0.0013. *P*-values were determined using the unpaired Student t test.

In *B. burgdorferi*-infected mice, absence of TLR signaling does not diminish the severity of arthritis and in fact, has even been reported to enhance inflammation (Bolz et al., 2004). In this situation, IgG opsonization of *B. burgdorferi* and/or its membrane blebs could enhance the uptake of spirochetes and their inflammatory components via activating FcγR, and promote release of inflammatory cytokines, similar to what we observed when MyD88^−/−^ macrophages were stimulated with OspC immune complexes *in vitro*. We therefore determined the effects of FcγR deficiency alone or in combination with MyD88 deficiency on *B. burgdorferi*-induced arthritis. The severity of arthritis was highest in MyD88^−/−^ mice at day 14, although FcεR_γ^−/−^_ mice also exhibited a modest increase in joint inflammation relative to WT at this time point (Figure 3A). At day 21, WT mice had increased arthritis in comparison to the day 14 timepoint, whereas the arthritis detected in MyD88^−/−^ was less pronounced. FcεR_γ^−/−^_ mice had the highest arthritis severity scores relative to WT or MyD88^−/−^ mice at 21 days of infection. Mice deficient in both FcγR and MyD88 had minimal arthritis at this time point (Figure 3B).

### Arthritis prevalence in the FcεR_γ^−/−^_ MyD88^−/−^ double knockout mice was lower than that of WT or single knockout mice early after infection

To assess the effects of FcγR deficiency on prevalence of arthritis after *B. burgdorferi* infection, we determined the number of joints that had evidence of inflammation in WT and MyD88^−/−^ mice for comparison with FcεR_γ^−/−^_ and the double knockout FcεR_γ^−/−^_ MyD88^−/−^ mice (Table 1). At day 14 of infection, the prevalence of arthritis was higher in MyD88^−/−^ and FcεR_γ^−/−^_ mice than in WT or FcεR_γ^−/−^_ MyD88^−/−^ mice (Table 1). The prevalence remained higher in each of the single knockout strains as compared to the WT mice at day 21. At this time point the arthritis in the double knockout mice was similar in prevalence to what had been observed in each of the single knockout mouse groups (Table 1).

**Table 1:** **Prevalence of arthritis in *B. burgdorferi* infected mic^a^**.

**Day of infection**	**Mouse group**			
	**WT**	**FcεR_γ−/−_**	**MyD88^−/−^**	**FcεR_γ−/−_MyD88^−/−^**
14	22/40	60/75^b^	36/44^c^	8/16
21	38/75	86/108^d^	46/58^e^	51/65^f^

a Arthritis prevalence was reported as the number of joints with inflammation over the total number of joints examined in each group. The day 14 data are the prevalence combined from two separate experiments, and at day 21 the data for the prevalence are combined from four experiments. Statistically significant differences were found between the following groups.

b FceR_γ^−/−^_ vs. WT P = 0.009 and vs. FceR_γ^−/−^_MyD88^−/−^ P = 0.02.

c MyD88^−/−^ vs. WT P = 0.01 and vs. FceR_γ^−/−^_MyD88^−/−^ P = 0.02.

d FceR_γ^−/−^_ vs. WT P ≤ 0.0001.

e MyD88^−/−^ vs. WT P = 0.001.

f FceR_γ^−/−^_MyD88^−/−^ vs. WT P = 0.0008. P-values were determined using the Fisher's exact test.

### MyD88^−/−^ mice exhibit a delay in the resolution of knee arthritis that is abrogated in the absence of FcγR

We examined *B. burgdorferi*-infected mice at a later time point, day 45, when arthritis is normally subsiding. Mild inflammation was still present in tibiotarsal joints from each mouse group at infection day 45 (Table 2). MyD88^−/−^ and WT mice exhibited comparable degrees of inflammation in these joints whereas arthritis scores were lower in FcεR_γ^−/−^_ mice and FcεR_γ^−/−^_ MyD88^−/−^ (Table 2). We found that arthritis in the knees was completely resolved in the WT and FcεR_γ^−/−^_ mice and a single joint in one FcεR_γ^−/−^_ MyD88^−/−^ mouse had a small amount of inflammation. This was not the case with MyD88^−/−^ mice, which had the highest prevalence of arthritis, with 8 of 10 knees showing some degree of inflammation (Table 2). No differences in carditis were observed at day 21 and carditis had resolved by day 45 (data not shown).

**Table 2:** **MyD88-deficient mice exhibit knee arthritis at day 45 of infection that is reduced in prevalence and severity in the absence of FcγR**.

**Mouse strain**	**Joints**	**Arthritis prevalence^a^**	**Arthritis severity^b^**
WT	Total	7/20	0.45 ± 0.8
	Tibiotarsal	7/10	0.9 ± 0.9
	Knees	0/10	0
FcεR_γ^−/−^_	Total	4/20	0.24 ± 0.6
	Tibiotarsal	4/10	0.48 ± 0.8
	Knees	0/10	0
MyD88^−/−^	Total	16/20^c^	0.8 ± 0.6^d^
	Tibiotarsal	8/10	1.1 ± 0.8^e^
	Knees	8/10^f^	0.58 ± 0.4^g^
FcεR_γ^−/−^_MyD88^−/−^	Total	7/20	0.18 ± 0.3
	Tibiotarsal	6/10	0.3 ± 0.3
	Knees	1/10	0.05 ± 0.16

a Arthritis prevalence was reported as the number of joints with inflammation over the total number of joints examined in each group.

b Arthritis was scored on a scale of 0 (negative) to 3 (severe) and reported as the average score of all the joints examined in each mouse group. Five mice were used in each group. Statistically significant differences were found between the following groups.

c MyD88^−/−^ vs. WT P = 0.01, vs. FcεR_γ^−/−^_ P = 0.004, and vs. FcεR_γ^−/−^_ MyD88^−/−^ P = 0.01.

d MyD88^−/−^ vs. WT P = 0.0.04, vs. FcεR_γ^−/−^_ P = 0.001, and vs. FcεR_γ^−/−^_ MyD88^−/−^ P = 0.0008.

e MyD88^−/−^ vs. FcεR_γ^−/−^_^−^MyD88^−/−^ P = 0.02.

f MyD88^−/−^ vs. WT P = 0.007, vs. FcεR_γ^−/−^_ P = 0.007, and vs. FcεR_γ^−/−^_ MyD88^−/−^ P = 0.006.

g FKO vsWT P = 0.04, vs. FcεR_γ^−/−^_ P = 0.001, and vs. FcεR_γ^−/−^_ MyD88^−/−^ P = 0.0008.

^c,f^Fisher's exact test.

^d,e,g^Mann-Whitney test.

### FcεR_γ^−/−^_MyD88^−/−^ double knockout mice have higher serum titers of anti-*B. burgdorferi* IgG3 antibodies than MyD88^−/−^ mice

**Figure 4:**
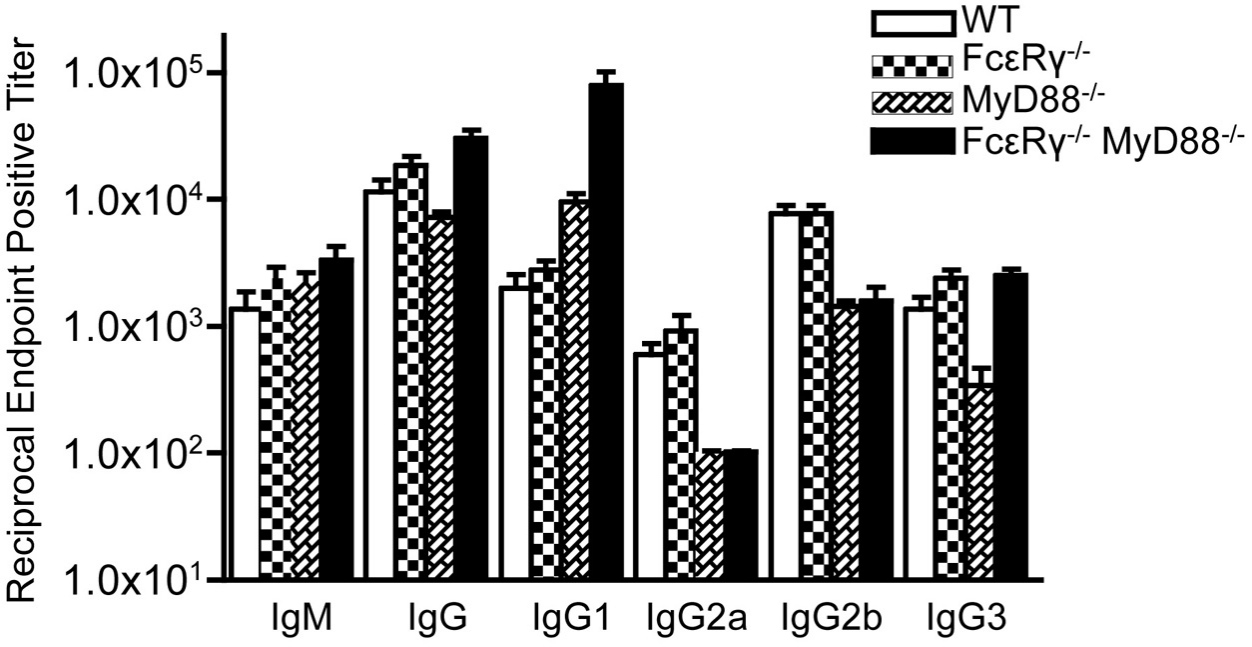
**Anti-*B. burgdorferi* IgG subclass titers are different in the single and double knockout mice**. Anti-*B. burgdorferi* specific titers were determined by ELISA and are reported as the reciprocal of the endpoint positive titers. The FcεR_γ^−/−^_ MyD88^−/−^ anti-*B. burgdorferi* IgG1 titers are significantly higher than the MyD88^−/−^ titers (*P* = 0.0113). The MyD88^−/−^ IgG 3 titers are significantly lower than the FcεR_γ^−/−^_ MyD88^−/−^ titers (*P* = 0.0025) as well as the WT and FcεR_γ^−/−^_ titers (*P* = 0.01 and.0025, respectively).

We have previously shown that *B. burgdorferi*-specific IgG in infected MyD88^−/−^ mice exhibit a shift toward the IgG1 subclass, although IgG2a, IgG2b, and IgG3 subclass responses can still be detected, albeit at lower levels (Liu et al., 2004). At 21 days of infection, FcεR_γ^−/−^_ mice had anti-*B. burgdorferi* IgG subclass responses similar to WT mice (Figure 4). MyD88^−/−^ mice, as expected, had reduced IgG2a and 2b titers, reduced IgG3 titers, and increased IgG1 titers compared to WT mice. The FcεR_γ^−/−^_MyD88^−/−^ double knockout mice also had reduced levels of *B. burgdorferi*-specific IgG2a and 2b, and elevated IgG1, similar to the single knockout MyD88^−/−^ mice (Figure 4). The *B. burgdorferi*-specific IgG3 levels in the double knockout mice however, were greater than titers in MyD88^−/−^ single knockout mice and similar to those of WT and FcεR_γ^−/−^_ mice (Figure 4).

### Gene expression profiles are altered in *B. burgdorferi* infected FcεR_γ^−/−^_MyD88^−/−^ mouse joints

To globally assess the cytokines that may be involved with arthritis in our mouse groups, we measured the changes in expression levels of select cytokine and chemokines in RNA isolated from knee joints using the RT^2^ Profiler PCR Array for Inflammatory Cytokines and Receptors (SA Biosciences). Each mouse group was analyzed compared to uninfected controls of the same phenotype (Table 3). Analysis of knee joint RNA demonstrated that FcεR_γ^−/−^_ MyD88^−/−^ mice exhibit a unique expression pattern that was not seen in either of the single knockouts or in WT mice. In WT mice, we found that the most abundantly up-regulated gene in infected joints was the B cell chemokine Cxcl13 (215 fold) (Table 4). It was also significantly upregulated in the FcεR_γ^−/−^_ mice (129 fold) and FcεR_γ^−/−^_ MyD88^−/−^ (40 fold) infected joints, but not in the joints of MyD88^−/−^ mice (0.39 fold). FcεR_γ^−/−^_ MyD88^−/−^ joints expressed less Cxcl1, Xcr 1, IL-1β, and C reactive protein than did the joint tissues from either single knockout or WT infected joints (Table 3). In order to confirm the results of the Superarray, RT-PCR reactions were performed on a select set of genes using the same RNA as was used on the array. Results using primers for TNFα, IL-10, and IFNγ show that the trends for increased or decreased expression or very low expression were consistent between the array and the PCR results (Table 4). The Superarray, however, was more sensitive than the PCR (Table 4).

**Table 3:** **Microarray analysis of inflammatory genes expressed in mouse knee joints after *B. burgdorferi* infection**.

**Gene**	**WT Inf/Un**	**MyD88^−/−^ Inf/Un**	**FcεR_γ−/−_ Inf/Un**	**MyD88^−/−^ FcεR_γ−/−_Inf/Un**	**MyD88^−/−^ ratio/ WT ratio**	**FcεR_γ−/−_ ratio/ WT ratio**	**MyD88^−/−^ FcεR_γ−/−_ratio/ WT ratio**
1-Ccl1	1.97	12.55	1.27	6.06	6.37	0.64	3.08
1-Ccl11	1.15	5.86	0.02	0.64	5.1	0.02	0.56
1-Ccl12	2.66	4.76	0.66	11.79	1.79	0.25	4.43
1-Ccl17	0.13	5.1	0.06	14.83	39.23	0.46	114.08
1-Ccl19	1.18	2.55	2.89	5.54	2.3	2.45	4.69
1-Ccl2	1.8	8.88	3.2	11.88	4.93	1.78	6.6
1-Ccl20	1.95	20.39	6.92	0.99	10.46	3.55	0.51
1-Ccl22	2.41	4.44	0.09	3.66	1.84	0.04	1.52
1-Ccl24	0.21	1.37	0.04	0.45	6.52	0.19	2.14
1-Ccl25	1.04	0.34	0.62	0.78	0.33	0.6	0.75
1-Ccl3	4.08	1.68	2.25	2.1	0.41	0.55	0.51
1-Ccl4	1.74	4.14	0.63	2.17	2.38	0.36	1.25
1-Ccl5	0.34	8.88	0.21	5.35	26.12	0.62	15.74
1-Ccl6	0.25	6.28	0.35	8.22	25.12	1.4	32.88
1-Ccl7	3.66	14.42	2.19	14.83	3.94	0.6	4.05
1-Ccl8	3.94	100.43	0.29	67.18	25.49	0.07	17.05
1-Ccl9	0.34	6.28	0.95	2.62	18.47	2.79	7.71
1-Cx3cl1	0.72	6.73	0.56	1.59	9.35	0.78	2.21
1-Cxcl1	5.46	2.55	2.1	0.54	0.47	0.38	0.1
1-Cxcl10	2.5	26.91	8.51	57.28	10.76	3.4	22.91
1-Cxcl11	N/A	8.88	0.29	5.1	N/A	N/A	N/A
1-Cxcl12	0.42	0.68	0.18	0.34	1.62	0.43	0.81
1-Cxcl13	215.27	0.39	128.89	40.5	0.002	0.6	0.19
1-Cxcl15	3.73	0.78	1.3	0.51	0.21	0.35	0.14
1-Cxcl9	0.75	26.91	0.98	22.47	35.88	1.31	29.96
2-Ccr1	0.2	3.36	0.11	0.59	16.8	0.55	2.95
2-Ccr2	N/A	2.93	0.07	0.69	N/A	N/A	N/A
2-Ccr3	0.63	5.1	0.1	0.68	8.1	0.16	1.08
2-Ccr4	0.75	1.46	0.47	1.67	1.95	0.63	2.23
2-Ccr5	1.12	38.05	0.07	1.16	33.97	0.06	1.04
2-Ccr6	1.03	0.84	0.05	0.66	0.82	0.05	0.64
2-Ccr7	0.32	0.9	0.15	0.33	2.81	0.47	1.03
2-Ccr8	0.54	15.45	0.31	0.95	28.61	0.57	1.76
2-Ccr9	0.8	1.04	0.04	0.16	1.3	0.05	0.2
2-Cxcr3	1.32	7.73	0.75	5.74	5.86	0.57	4.35
2-Il8rb	0.37	0.32	0.04	0.17	0.86	0.11	0.46
2-Xcr1	13.74	0.97	0.23	0.15	0.07	0.02	0.01
3-1-Il13	4.56	7.73	0.26	0.51	1.7	0.06	0.11
3-Cd40lg	1.09	1.46	0.04	0.7	1.34	0.04	0.64
3-Ifng	0.8	10.93	0.86	8.82	13.66	1.08	11.03
3-Il10	2.27	530.06	4.38	6.15	233.51	1.93	2.71
3-Il11	0.41	2.22	0.2	0.41	5.41	0.49	1
3-Il15	0.39	3.14	0.19	0.48	8.05	0.49	1.23
3-Il16	0.1	0.78	0.02	0.11	7.8	0.2	1.1
3-Il17b	0.34	1.04	0.23	0.25	3.06	0.68	0.74
3-Il18	0.56	0.84	0.86	1.01	1.5	1.54	1.8
3-Il1a	1.59	12.55	0.96	1.65	7.89	0.6	1.04
3-Il1b	5.74	3.36	1.21	0.86	0.59	0.21	0.15
3-Il1f6	2.45	1.04	2.99	1.34	0.42	1.22	0.55
3-Il1f8	1.41	0.84	1.26	0.46	0.6	0.89	0.33
3-Il20	2.13	0.84	6.41	1.21	0.39	3.01	0.57
3-Il3	1.27	0.84	4.23	1.05	0.66	3.33	0.83
3-Il4	1.06	14.42	0.33	2.51	13.6	0.31	2.37
3-Itgam	1.44	9.51	0.67	0.58	6.6	0.47	0.4
3-Itgb2	N/A	10.2	3.36	2.81	N/A	N/A	N/A
3-Lta	0.74	0.26	0.97	0.55	0.35	1.31	0.74
3-Ltb	0.31	0.52	0.03	0.24	1.68	0.1	0.77
3-Mif	0.58	2.55	0.41	0.93	4.4	0.71	1.6
3-Scye1	N/A	0.97	0.64	0.81	N/A	N/A	N/A
3-Spp1	0.93	0.24	0.66	0.2	0.26	0.71	0.22
3-Tgfb1	0.16	0.9	0.1	0.27	5.63	0.63	1.69
3-Tnf	0.32	16.56	0.25	0.26	51.75	0.78	0.81
4-Il10ra	0.16	5.46	0.22	0.53	34.13	1.38	3.31
4-Il10rb	N/A	4.44	0.37	0.57	N/A	N/A	N/A
4-Il13ra1	0.82	2.38	0.58	1.36	2.9	0.71	1.66
4-Il1r1	N/A	2.38	0.32	0.41	N/A	N/A	N/A
4-Il1r2	1.85	0.78	1.36	0.99	0.42	0.74	0.54
4-Il2rb	0.18	2.22	0.02	0.45	12.33	0.11	2.5
4-Il2rg	0.29	10.93	0.15	1.03	37.69	0.52	3.55
4-Il5ra	0.99	0.45	0.09	0.44	0.45	0.09	0.44
4-Il6ra	0.56	2.73	0.21	0.74	4.88	0.38	1.32
4-Il6st	0.41	2.07	0.1	0.27	5.05	0.24	0.66
4-Tnfrsf1a	0.03	3.61	0.03	0.07	120.33	1	2.33
4-Tnfrsf1b	0.27	5.46	0.23	0.88	20.22	0.85	3.26
5-Abcf1	0.64	1.27	0.43	0.59	1.98	0.67	0.92
5-Bcl6	0.18	2.55	0.08	0.19	14.17	0.44	1.06
5-C3	0.68	19.03	0.1	2.55	27.99	0.15	3.75
5-Casp1	3.73	1.8	2.1	3.41	0.48	0.56	0.91
5-Crp	1.75	1.11	3.58	0.51	0.63	2.05	0.29
5-Cxcl5	1.12	0.28	0.57	0.3	0.25	0.51	0.27
5-Tollip	0.26	4.14	0.31	0.38	15.92	1.19	1.46
Actb	0.16	2.38	0.26	0.65	14.88	1.63	4.06
Ccr10	0.66	2.22	0.04	0.1	3.36	0.06	0.15
Cxcr5	1.01	0.3	0.14	0.66	0.3	0.14	0.65
Gapdh	0.2	1.68	0.09	0.22	8.4	0.45	1.1
Gusb	0.72	8.28	0.71	1.2	11.5	0.99	1.67
Hprt1	1.41	1.19	2.16	1.74	0.84	1.53	1.23
Hsp90ab1	0.71	0.84	0.65	0.48	1.18	0.92	0.68
Pf4	0.14	0.9	0.23	0.2	6.43	1.64	1.43

PCR-array PAMM—011 (SABiosciences). Values shown in the first four columns are ratios of ΔΔ ct values from uninfected and infected joints from each strain (pooled RNA from joints of four mice per group) calculated using the SABiosciences template for analysis. The last three columns show ratios comparing WT ratios to knockout ratios. The five genes at the end of the table, Gapdh, Gusb, Hprt1, Hsp90ab1, Pf4 are housekeeping genes. Ratios >2 or <0.5 are considered significant in this assay.

**Table 4:** **Detection of select cytokines by RT-PCR in 21-day infected mouse joints**.

**Mouse strain**	**TNFα**	IL-10^*^	**IFNγ**
WT Inf/Uninf	0.4	0.0.35	N/A
MyD88^−/−^ Inf/Uninf	136.4	3.5	4.9
FcϵR_γ^−/−^_ Inf/Uninf	3.5	1.3	0.13
FcϵR_γ^−/−^_ MyD88^−/−^ Inf/Uninf	N/A	N/A	15.6

RT-PCR was performed using the same RNA that was isolated for the PCR Super arrays. The amount of PCR product was estimated from the density of the bands in the agarose gels using ImageJ software. The data reported are the ratio of density values for Infected sample/Infected tubulin control to Uninfected sample/Uninfected tubulin controls. N/A, not available.

* The levels of IL-10 PCR product were high for all of the samples thus the magnitude of the differences calculated by ImageJ are lower than what was observed in the PCR array.

## Discussion

*B. burgdorferi* lipoproteins have been shown to activate innate immune cells *in vitro* through interaction with TLR that signal the secretion of pro-inflammatory cytokines and chemokines (Weis and Bockenstedt, 2010). As deficiency in TLR2 or its intracellular signaling molecule MyD88 is sufficient to eliminate *B. burgdorferi* lipoprotein-induced innate immune cell activation, the observation that arthritis still occurs in TLR2- and MyD88-deficient mice suggests involvement of other inflammatory pathways. Here, we determined the contribution of FcγR to host defense and disease associated with *B. burgdorferi* infection. Our findings in *B. burgdorferi*-infected FcεR_γ^−/−^_ mice reveal a modest role for FcγR in limiting pathogen burden early in infection, with spirochete numbers about 10-fold higher than those found in WT mice. This elevated pathogen burden was transient and associated with enhanced inflammation in joints at day 14 after infection, but not thereafter. In contrast, in later stages of infection when disease is resolving, our results suggest that FcγR may promote arthritis in MyD88^−/−^ mice, as elimination of activating FcγR in MyD88^−/−^ mice significantly attenuates this disease manifestation.

FcγR are thought to bridge innate and adaptive immunity through the relative engagement of activating and inhibitory FcγR. The subclasses of IgG produced have different binding affinities for FcγR, which influences the degree of inflammatory cytokine secretion (Nimmerjahn and Ravetch, 2006). IgG2a antibodies are the most potent at activating cells and preferentially bind activating FcγRI, whereas IgG1 antibodies are less activating and bind to low affinity FcγRIII. Our analysis of *B. burgdorferi*-specific IgG subclasses showed no significant difference between WT and FcεR_γ^−/−^_ mouse groups, so arthritis susceptibility at 14 days of infection cannot be attributed to a shift toward a more inflammatory IgG subclass. In murine Lyme borreliosis, IgG is detectable within a week or so of infection and may serve to opsonize *B. burgdorferi* and augment phagocyte clearance of the pathogen. In FcεR_γ^−/−^_ mice, phagocytes may rely on TLR/MyD88-dependent pathways for disposal of *B. burgdorferi*, with secondary consequences of enhanced inflammatory cytokine secretion and arthritis in the early stages of infection. A similar reliance on TLR/MyD88-dependent responses for pathogen clearance may explain the effects of complement component C3 deficiency on the course of murine Lyme borreliosis (Lawrenz et al., 2003). Activation of complement by the classical, lectin, or alternative pathways results in deposition of C3b on the bacterial surface, enhancing its killing via C3b-mediated phagocytosis or by membrane attack complex formation. C3^−/−^ mice infected with *B. burgdorferi* harbor ~3-fold more spirochetes in joints in comparison to wild type mice and exhibit a trend toward more severe arthritis, which could be due to activation of TLRs (Lawrenz et al., 2003).

We and others have previously reported that *B. burgdorferi*-infected MyD88^−/−^ mice have markedly elevated pathogen burdens, but paradoxically, the degree of arthritis is similar to that observed in WT mice (Bolz et al., 2004; Liu et al., 2004). MyD88^−/−^ mice have a shift in *B. burgdorferi*-specific IgG subclass to IgG1. Opsonization of spirochetes or their remnants with this antibody subclass will preferentially activate FcγRIII, which in many model systems is negatively regulated by FcγRIIb (Nimmerjahn and Ravetch, 2008). This pattern of FcγR engagement in MyD88^−/−^ mice may provide one explanation for the lack of correlation between the high pathogen burden and disease. In addition, MyD88^−/−^ mice express higher levels of the anti-inflammatory cytokine IL-10, which could also lead to reduced arthritis in the setting of elevated pathogen burdens (Wooten et al., 2002).

Our studies using FcεR_γ^−/−^_ MyD88^−/−^ mice have unveiled a contribution of FcγR and immune complexes to disease. *In vitro*, immune complexes can stimulate MyD88^−/−^ macrophages to produce TNFα, supporting the possibility that FcγR contribute to arthritis in MyD88^−/−^ mice. *In vivo* studies with FcεR_γ^−/−^_ MyD88^−/−^ double knockout mice reveal lower arthritis severity scores than either of the single knockout strains or in WT mice, suggesting that both FcγR and MyD88-dependent pathways may contribute to the development of arthritis, and each pathway may take on a more prominent role when the other is inactivated. The dominance of TLR/MyD88-dependent inflammation over lipidation-independent signaling via FcγR is supported by an earlier report that *B. burgdorferi*-induced TNFα production by bone-marrow derived macrophages from FcεR_γ^−/−^_ mice was not significantly reduced compared to wild type macrophages (Talkington and Nickell, 2001).

To gain further insight into the immune molecules involved in arthritis development when FcγR or MyD88-dependent pathways were interrupted, genes expressed in joints from 21 days infected mice were determined using microarray and compared to uninfected control mice of the same genotype. We found differences in genes expressed among the uninfected control WT and the single and double knockout control mice, and each mouse group exhibited additional differences after infection. More striking differences were noted between WT and MyD88^−/−^ mice than between WT and FcεR_γ^−/−^_ mice. The FcεR_γ^−/−^_ MyD88^−/−^ mice showed decreased expression of several proteins known to be involved in inflammation as compared to WT and either of the single knockout mice. The most strongly upregulated gene in the joints of all the strains except for the MyD88^−/−^ mice was Cxcl13, a B cell chemoattractant cytokine. Cxcl13 is significantly upregulated in the cerebrospinal fluid of patients with acute neuroborreliosis and may serve as a diagnostic marker for disease (Senel et al., 2010a,b). It has also been found that Cxcl13 mRNA levels are elevated in synovial tissues from rheumatoid arthritis patients, but not in osteoarthritic joints (Shi et al., 2001; Carlsen et al., 2004; Meeuwisse et al., 2011). Cxcl13 may mediate recruitment of immune cells to infected joints during infection, but since the MyD88^−/−^ mice (which have reduced expression of Cxcl13) still develop arthritis, this suggests that other chemokines may also play significant roles. We did not isolate and quantify the cell populations in the joints, but histologically the predominant infiltrate in the inflamed joints was neutrophilic (data not shown). The MyD88^−/−^ mice had large increases in Ccl8 expression (100 fold) and Cxcl 9 and 10 (both 27 fold). The reduced levels of arthritis incidence and severity in the FcεR_γ^−/−^_ MyD88^−/−^ mice correlated with reduced expression of several chemokines and cytokines compared to the three other strains. Cxcl1, reduced in FcεR_γ^−/−^_ MyD88^−/−^ mice, functions to recruit neutrophils (Coelho et al., 2008; Conte et al., 2008; Grespan et al., 2008; Ritzman et al., 2010), which are a main component of the cell infiltrates found in arthritic joints of the infected mice. A decrease in expression of Cxcl1 (also known as KC) may reduce neutrophil recruitment. WT and single knockout mice have modest increases in Cxcl1 (ranging from 2–5.5 fold), which may be sufficient in conjunction with other chemoattractants to recruit cells to the joint. IL-1β levels were also found to be lower in the FcεR_γ^−/−^_ MyD88^−/−^ mice. This may not play a major role in the reduced disease, however, as we have previously shown that caspase 1, which cleaves IL-1β into its active form, is not essential for the control of disease (Liu et al., 2009). The chemokine receptor Xcr1 was also selectively down regulated in the FcεR_γ^−/−^_ MyD88^−/−^ mice. Xcr1 is the receptor for Xcl1 and Xcl2, which were not part of our microarray, but it is known that Xcrl also plays a role in rheumatoid arthritis (Wang et al., 2004) and in the recruitment of cells to arthritic joints (Coelho et al., 2008; Grespan et al., 2008). Xcr1 was upregulated in the WT mice (13.7 fold) but not in the FcεR_γ^−/−^_ or MyD88^−/−^ mice. Taken together, these results suggest that multiple chemokines and their receptors contribute to the development of murine Lyme arthritis, and that the molecules involved are similar to those seen in other forms of antigen-induced and autoimmune-mediated arthritis.

In summary, our studies reveal a role for FcγR in limiting pathogen burden and disease in mice early after infection with the extracellular pathogen *Borrelia burgdorferi*. We also show that FcγR may contribute to prolonged arthritis in certain settings, such as in MyD88^−/−^ mice when phagocytosis is impaired and pathogen burden is high. Polymorphisms in FcγR have been found in humans that affect IgG subclass binding and responses (Kapur et al., 2014), which could influence the outcome from Lyme disease. Additional studies are needed to determine whether FcγR and/or polymorphisms in these receptors affect the expression of human Lyme arthritis.

### Conflict of interest statement

The authors declare that the research was conducted in the absence of any commercial or financial relationships that could be construed as a potential conflict of interest.
